# Intravitreal Clindamycin and Dexamethasone Combined with Systemic Oral Antitoxoplasma Therapy versus Intravitreal Therapy Alone in the Management of Toxoplasma Retinochoroiditis: A Retrospective Study

**DOI:** 10.1155/2018/4160837

**Published:** 2018-02-12

**Authors:** Ashraf Bor'i, Ashraf Mahrous, Mahmoud A. Al-Aswad, Haitham Y. Al-Nashar, Waled M. Nada, Mostafa Wagih, Ahmed M. B. Awad, Wael M. El-Haig

**Affiliations:** Department of Ophthalmology, Zagazig University, Zagazig, Egypt

## Abstract

**Purpose:**

To compare clinical outcome of IVCD combined with oral therapy with IVCD alone in patients with toxoplasmic retinochoroiditis.

**Patients and Methods:**

Thirty eyes were reviewed. Two equal groups were identified (15 eyes each). Clinical outcome measures were resolution of active inflammation, changes in BCVA and CMT, adverse drug reactions, and rate of recurrence.

**Results:**

Mean baseline of BCVA 1.08 ± 0.17 and 1.03 ± 0.15 improved to 0.64 ± 0.18 and 0.69 ± 0.17 at the end of follow-up in group I and II, respectively. No statistically significant difference was observed. CMT was 392.6 ± 33.16 *μ*m and 397.3 ± 14.6 *μ*m significantly decreased to 314.7 ± 4.43 *μ*m and 319.6 ± 7.8 *μ*m. Resolution of acute inflammation was achieved in all cases in both groups. There were no recurrent cases in group I, and only one out of 15 (6.7%) in group II. No ocular or systemic adverse events were recorded.

**Conclusion:**

IVCD is an effective route of treatment for active toxoplasmic retinochoroiditis that can be used solely without the need to use systemic medications..

## 1. Introduction

Toxoplasmosis, caused by *Toxoplasma gondii* (*T. gondii*), is the most common infection of the retina in general populations worldwide.

*T. gondii* infection is responsible for the majority of infectious uveitis cases [1, 2]. This disease typically affects the posterior pole of one eye, and lesions can present as either solitary, multiple, or satellite in nature, or as a pigmented retinal scar. Active lesions present as gray-white foci of retinal necrosis with adjacent choroiditis, vasculitis, hemorrhage, and vitritis. Cicatrization progresses from the periphery towards the center, with variable pigmentary changes. Anterior uveitis is another common finding, presenting with mutton-fat keratic precipitates, anterior chamber cells and flare, and posterior synechiae [3–5].

The diagnosis of ocular toxoplasmosis typically depends on clinical findings. When clinical diagnosis through fundus examination cannot be established with certainty, polymerase chain reaction could be ordered to look for antitoxoplasmosis antibody in ocular fluids. This test has shown high specificity and sensitivity to confirm the diagnosis [6, 7].

Ocular toxoplasmosis therapy includes the administration of antimicrobial drugs with or without the use of corticosteroids. Several drugs have been proposed for treatment, including pyrimethamine, sulfadiazine, spiramycin, clindamycin, and trimethoprim-sulfamethoxazole [8].

The most common side effects were associated with pyrimethamine medication and included hematologic complications, such as thrombocytopenia and leucopenia, despite folinic acid supplementation [9].

Intravitreal clindamycin injection and possibly steroids may be indicated for patients who have contraindications for systemic therapy specific against toxoplasmosis [10, 11].

The aim of this study was to compare the efficacy of intravitreal clindamycin and dexamethasone (IVCD) plus systemic oral antitoxoplasma therapy against that of intravitreal therapy alone in the treatment of active toxoplasmic retinochoroiditis.

## 2. Patients and Methods

This retrospective study reviewed the hospital records of thirty patients with active ocular toxoplasmic retinochoroiditis diagnosed and received treatment during the period starting from May 2014 to June 2017. The study was carried out according to the WMA Declaration of Helsinki—Ethical Principles for Medical Research Involving Human Subjects. All cases were clinically diagnosed as active toxoplasmic retinochoroiditis. Measurement of best corrected visual acuity (BCVA) and colored fundus photography were done for all patients. Both fundus fluorescein angiography and spectral domain optical coherence tomography (Spectralis, Heidelberg Engineering GmbH) studies were performed if the ocular media were sufficiently clear for imaging.

**Figure 1:**
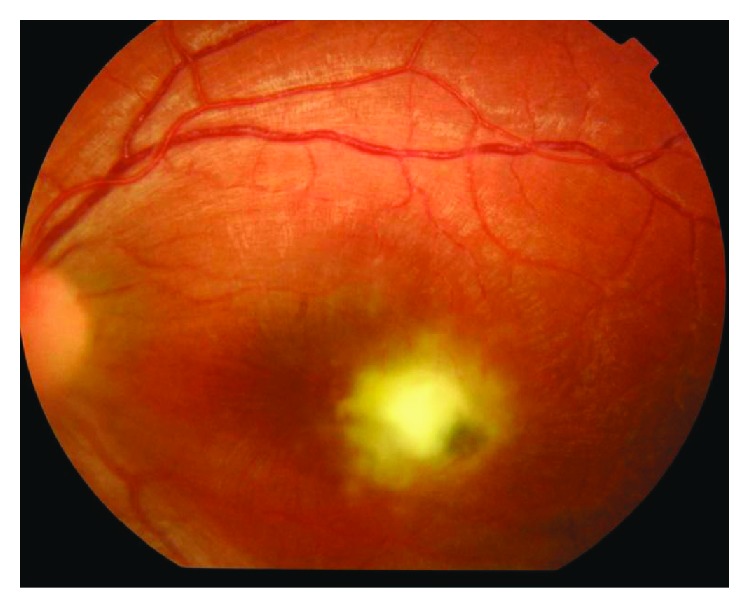
Group I (pretreatment).

**Figure 2:**
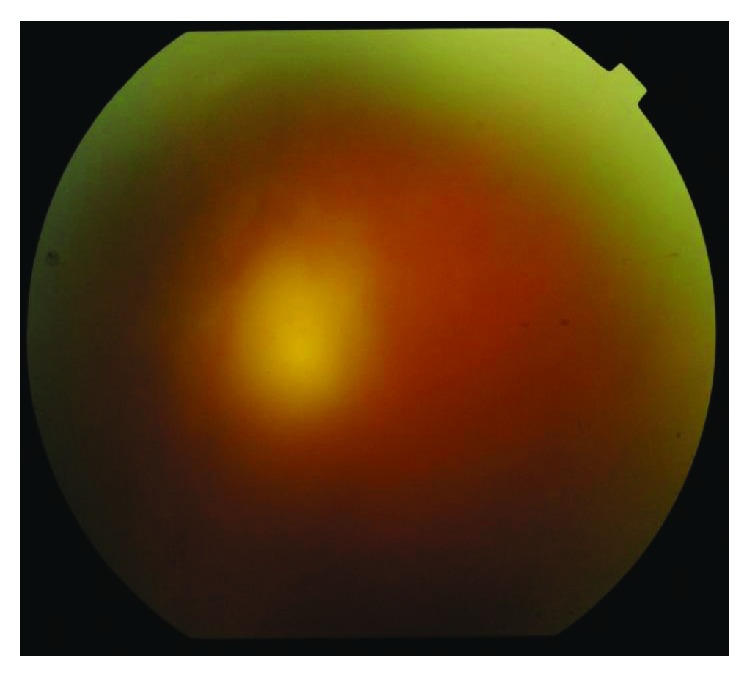
Group II (pretreatment).

Two groups of patients, with active toxoplasmic chorioretinitis in retinal zone 1, were identified. 15 eyes (15 patients) received treatment in the form of intravitreal injection combined with systemic treatment—group I (Figure 1). 15 eyes (15 patients) received intravitreal treatment alone without any systemic treatment—group II (Figure 2). Combination therapy was administered in group I with the intent to maximize the therapeutic effect.

Intravitreal injections were done under complete aseptic conditions. Two drugs were injected: clindamycin at a concentration of 1 mg/0.1 ml and dexamethasone at a concentration of 400 *μ*g/0.1 ml.

Patients in group I received systemic multidrug therapy combined with intravitreal treatment. The systemic multidrug therapy included pyrimethamine, sulfadiazine, and prednisolone.

Pyrimethamine was taken as a loading dose of 100 mg on the first day and followed by a dose of 25 mg, twice daily, for 6 weeks. Sulfadiazine was taken as a dose of 1 g, 4 times daily for 6 weeks. Prednisolone was taken as a dose of 60 mg/day for 2 weeks and then was tapered gradually.

Patient data at 3, 6, and 12 months after initiation of treatment were collected. The main clinical outcome measures were resolution of active inflammation, changes in BCVA expressed in LogMAR units, changes in central macular thickness measured by OCT, adverse drug reactions, and the rate of recurrence of the inflammation during follow-up.

Statistical analysis of the data was performed using the Student *t*-test, and *p* value determination (*p* value <0.05 was considered significant) and (*p* value >0.05 was considered insignificant).

## 3. Results

Thirty eyes of thirty patients with active toxoplasmic retinochoroiditis were treated either by intravitreal and systemic medications (15 eyes of 15 patients) or by only intravitreal medications (15 eyes of 15 patients).

The demographic data revealed that the mean age was 36.35 ± 6.29 years in group I and 35.55 ± 4.19 years in group II (*p* = 0.36); male to female ratio was 1.33 in group I and 1.5 in group II which was statistically insignificant.

In group I, mean baseline BCVA was 1.08 ± 0.17 LogMAR units; it improved to 0.8 ± 0.19 at the 3rd-month follow-up visit and to 0.72 ± 0.11 and 0.64 ± 0.18 at the 6th-month and the 12th-month follow-up visits, respectively. The difference was statistically significant at the last follow-up visit as compared to baseline value (*p* < 0.05).

In group II, mean baseline BCVA was 1.03 ± 0.15 LogMAR units, BCVA values improved to 0.87 ± 0.21 at the 3rd-month follow-up visit and to 0.75 ± 0.09 and 0.69 ± 0.17 at the 6th-month and the 12th-month visits, respectively. The difference was statistically significant at the last follow-up visit as compared to the pretreatment BCVA (*p* < 0.05).

**Figure 3:**
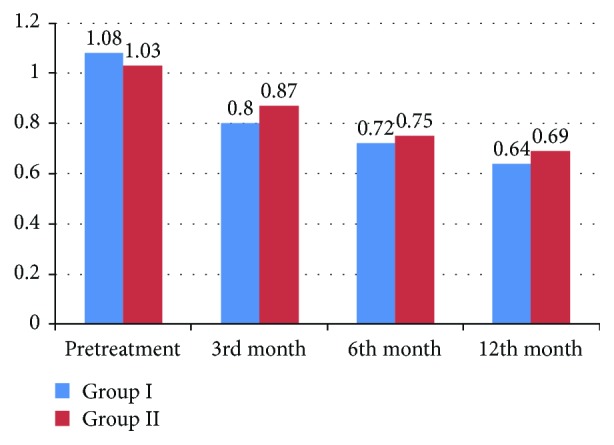
BCVA measured by LogMAR in patients of both groups.

No statistically significant difference was observed between group I and group II (*p* > 0.05) during the follow-up period (Table 1; Figure 3).

In group I, the mean central macular thickness (CMT) before treatment was 392.6 ± 33.16 *μ*m while in group II, it was 397.3 ± 14.6 *μ*m (no statistically significant difference, *p* > 0.05).

In group I, CMT decreased to 332.7 ± 8.2 *μ*m at the 3rd-month of follow-up visit and to 321.8 ± 11.2 *μ*m and 314.7 ± 4.43 *μ*m at the 6th-month and the 12th-month visits, respectively. The difference was statistically significant at the last follow-up visit as compared to the baseline thickness (*p* < 0.05).

While in group II, CMT improved to 340.9 ± 5.2 *μ*m at the 3rd-month of follow-up visit and to 325.9 ± 15.6 *μ*m and 319.6 ± 7.8 *μ*m at the 6th-month and the 12th-month visits, respectively. The difference was statistically significant at the last follow-up visit as compared to the baseline thickness (*p* < 0.05).

**Figure 4:**
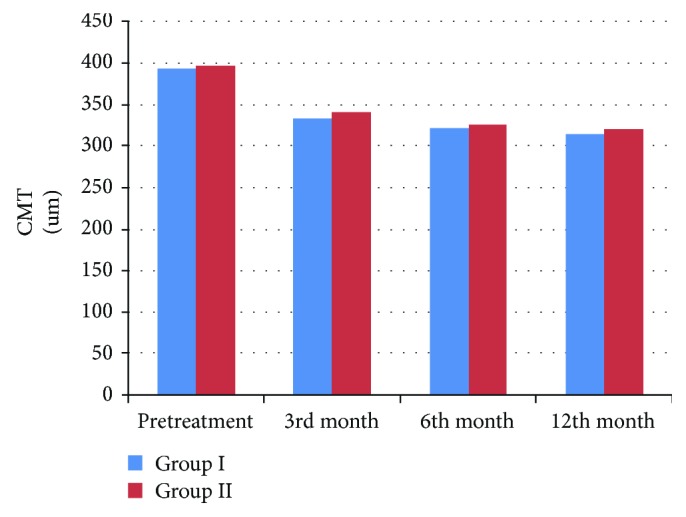
CMT measured by OCT in both groups.

There was no statistically significant difference observed between group I and group II (*p* > 0.05) during the follow-up period (Table 2; Figure 4).

**Figure 5:**
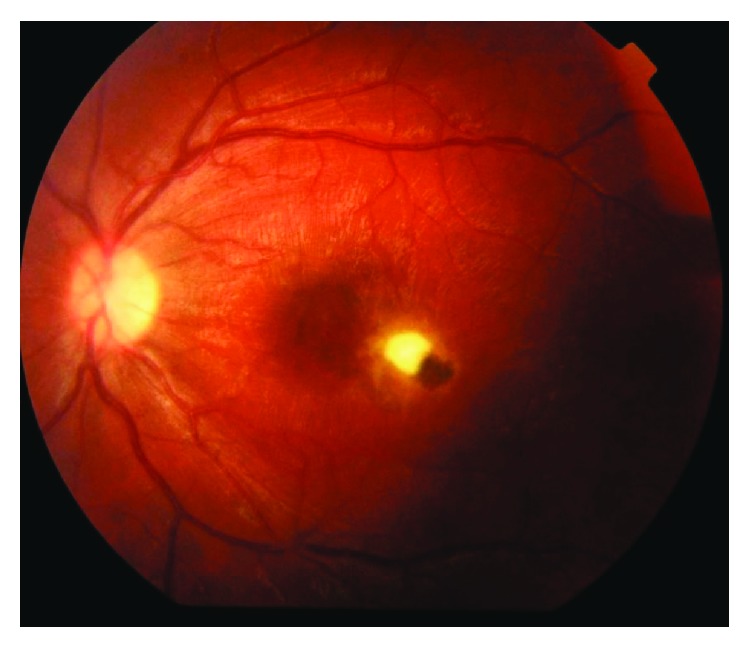
Group I (posttreatment).

**Figure 6:**
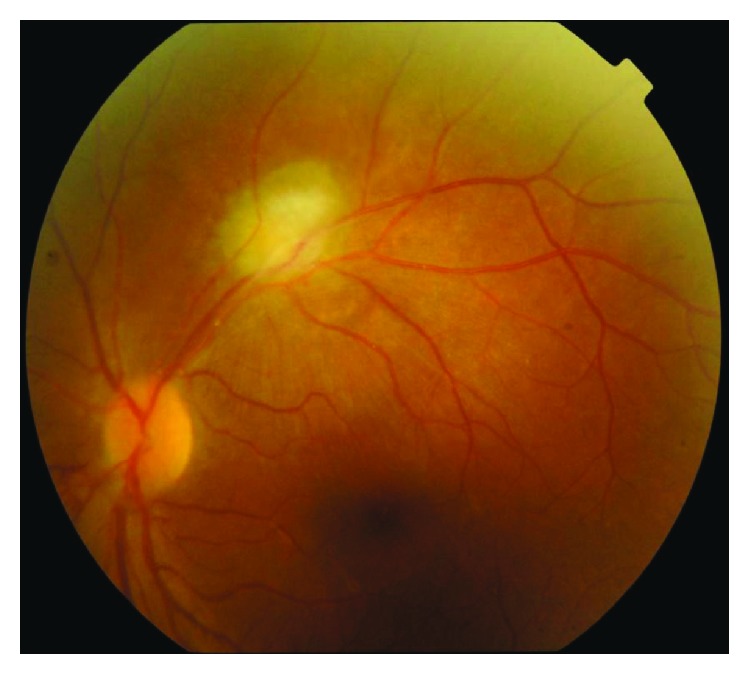
Group II (posttreatment).

Resolution of acute inflammation was achieved in all cases in both groups (Figures 5 and 6). To control the intraocular inflammation, the mean number of injections was 1.4 (range: 1–3 injections) with a mean interval of 19.4 ± 4.1 days in group I and the mean number of injections was 1.7 (range: 1–4 injections), with a mean interval of 17.6 ± 3.2 days in group II. There was no statistically significant difference observed between group I and group II during the follow-up period (*p* = 0.3).

The incidence of recurrent toxoplasmic retinochoroiditis in patients during the follow-up period was 0 out of 15 (0%) in group I and 1 out of 15 (6.7%) in group II. There was no statistically significant difference observed between group I and group II during the follow-up period (*p* = 0.2).

No ocular or systemic adverse events were observed in both groups.

## 4. Discussion

Toxoplasmosis is the most common cause of intraocular inflammation in the world. Ocular toxoplasmosis is a potentially blinding condition and is associated with severe morbidity [12]. Ocular toxoplasmosis therapy includes the administration of antimicrobial drugs with or without corticosteroids. Several drugs have been proposed for treatment including pyrimethamine, sulfadiazine, spiramycin, clindamycin, and trimethoprim-sulfamethoxazole [13].

Compliance remains an important issue, as the treatment often requires many pills daily. Monitoring of blood cell counts is mandatory during the treatment, and patients must be aware of the possible allergic reactions or other complications of systemic treatment [14].

The present study compared the combined intravitreal and systemic treatment with intravitreal injection alone in patients with ocular toxoplasmosis. Thirty eyes of thirty patients with active toxoplasmic retinochoroiditis were retrospectively reviewed. Two groups of patients were identified: group I was treated with a combined intravitreal and systemic therapy regimen, while group II was treated with intravitreal injection alone. All patients were followed up for 12 months.

In group I, the mean baseline BCVA improved from 1.08 ± 0.17 before the treatment to 0.64 ± 0.18 at the 12th-month follow-up visit. In group II, the mean baseline BCVA improved from 1.03 ± 0.15 to 0.69 ± 0.17, with no significant difference observed between the two groups at the end of the follow-up period.

These data are in agreement with Soheilian et al., [15] who compared the efficacy of the classical treatment of ocular toxoplasmosis (pyrimethamine, sulfadiazine, and prednisolone) with a regimen consisting of trimethoprim/sulfamethoxazole (cotrimoxazole) plus prednisolone. They found that BCVA after treatment was 0.12 (LogMAR) in the classical treatment group and 0.09 (LogMAR) in the trimethoprim/sulfamethoxazole treatment group, with no significant difference observed (*p* = 0.56).

Lasave et al., [16] reported the anatomic and functional outcomes of intravitreal clindamycin and dexamethasone treatment of toxoplasmic retinochoroiditis. They reported that the baseline BCVA improved from a value of 1 ± 0.4 to 0.5 ± 0.4 at the end of the follow-up period. They also reported a significant reduction in CMT by OCT from 387.6 ± 70.1 *μ*m to 185.2 ± 44.7 *μ*m after a 24-month follow-up period. They observed that resolution of toxoplasmic retinochoroiditis was achieved in all cases with a mean number of injections of 3.6 (range = 2–5 injections) and a mean interval of 15.5 ± 4 days. In comparison to the present study, the mean CMT before treatment was 392.6 ± 33.16 *μ*min in group I and 397.3 ± 14.6 *μ*min in group II, and those measurements decreased to 314.7 ± 4.43 *μ*m in group I and to 319.6 ± 7.8 *μ*m in group II at the 12th-month follow-up visits. There is no significant difference between the groups during the follow-up period. Resolution of acute inflammation was achieved in all cases and in both groups with a mean number of injections of 1.4 in group I and 1.7 in group II (*p* = 0.3).

Choudhury et al., [17] studied the role of intravitreal trimethoprim/sulfamethoxazole in the treatment of toxoplasmic retinochoroiditis and found that there is no evidence of retinal toxicity in all patients. They concluded that the combined intravitreal trimethoprim/sulfamethoxazole and dexamethasone treatment might be an alternative strategy in patients with toxoplasmic retinochoroiditis.

Felix et al. [18] compared the effects of trimethoprim-sulfamethoxazole treatment versus a placebo in reducing the risk of toxoplasmic retinochoroiditis recurrences. They found that the incidence of recurrence within 12 months was 0 out of 46 (0%) and 6 out of 47 (12.80%) in the trimethoprim-sulfamethoxazole and placebo-treated groups, respectively (*p* = 0.26). These data are comparable with this study where the incidence of toxoplasmic retinochoroiditis recurrence within 12 months of follow-up was 0 out of 15 (0%) in group I and 1out of 15 (6.7%) in group II with no significant difference observed between the two groups (*p* = 0.2).

## 5. Conclusions

It can be concluded from this study that repeated intravitreal injection of clindamycin and dexamethasone is an effective regimen of treatment for active toxoplasmic retinochoroiditis, sparing patients from the burden of daily intake of multiple oral medications and their possible adverse effects. However, this study has the limitation of being retrospective wherein the baseline state, the intervention, and outcome were obtained from already existing data.

## Figures and Tables

**Table 1:** BCVA (LogMAR) in both groups.

	Pretreatment	3rd month	6th month	12th month
Group I	1.08 ± 0.17	0.80 ± 0.19	0.72 ± 0.11	0.64 ± 0.18
Group II	1.03 ± 0.15	0.87 ± 0.21	0.75 ± 0.09	0.69 ± 0.17
*p* value	0.8	0.7	0.5	0.8

**Table 2:** Central macular thickness (CMT) measured by OCT in both groups.

	Pretreatment	3rd month	6th month	12th month
Group I	392.6 ± 33.16	332.7 ± 8.2	321.8 ± 11.2	314.7 ± 4.43
Group II	397.3 ± 14.6	340.9 ± 5.2	325.9 ± 15.6	319.6 ± 7.8
*p* value	0.6	0.5	0.5	0.7
